# Analysis of dermoscopic characteristic for the differential diagnosis of palmoplantar psoriasis and palmoplantar eczema

**DOI:** 10.1097/MD.0000000000023828

**Published:** 2021-02-05

**Authors:** Xiaoping Yu, Guo Wei, Chunchun Shao, Mingjie Zhu, Shuna Sun, Xiaojie Zhang

**Affiliations:** a First Clinical Medical College, Shandong University of Traditional Chinese Medicine; b Department of Dermatology, The Second Hospital of Shandong University; c Center of Evidence-based Medicine, Institute of Medical Sciences, the Second Hospital of Shandong University, Jinan, Shandong, China; d Colorado School of Public Health, University of Colorado, Colorado, United States; e Department of Dermatology, The Affiliated Hospital of Shandong University of Traditional Chinese Medicine, Jinan, Shandong, China.

**Keywords:** dermoscopy, eczema, inflammatory skin diseases, palmoplantar skin diseases, psoriasis

## Abstract

Dermoscopy is a noninvasive diagnostic technique that is of great value for the differential diagnosis of palmoplantar psoriasis and palmoplantar eczema. Considering the particularity of palmoplantar anatomy, the dermoscopic features of psoriasis and eczema in palm region show fewer differences, compared with those in other parts of the body. Only a few studies have examined the palmoplantar region of psoriasis and eczema patients under a dermoscope.

A total of 26 patients with palmoplantar psoriasis and 31 patients with palmoplantar eczema were enrolled in our study. Target palmoplantar areas were observed through general observation and under dermoscope.

We found that the presence of white scales and a regular arrangement of dots and globular vessels were significantly indicative of palmoplantar psoriasis, while yellowish scales and an irregular arrangement of atypical vessels were significantly indicative of palmoplantar eczema.

## 1 Introduction

### 1.1 Background

**Figure 1:**
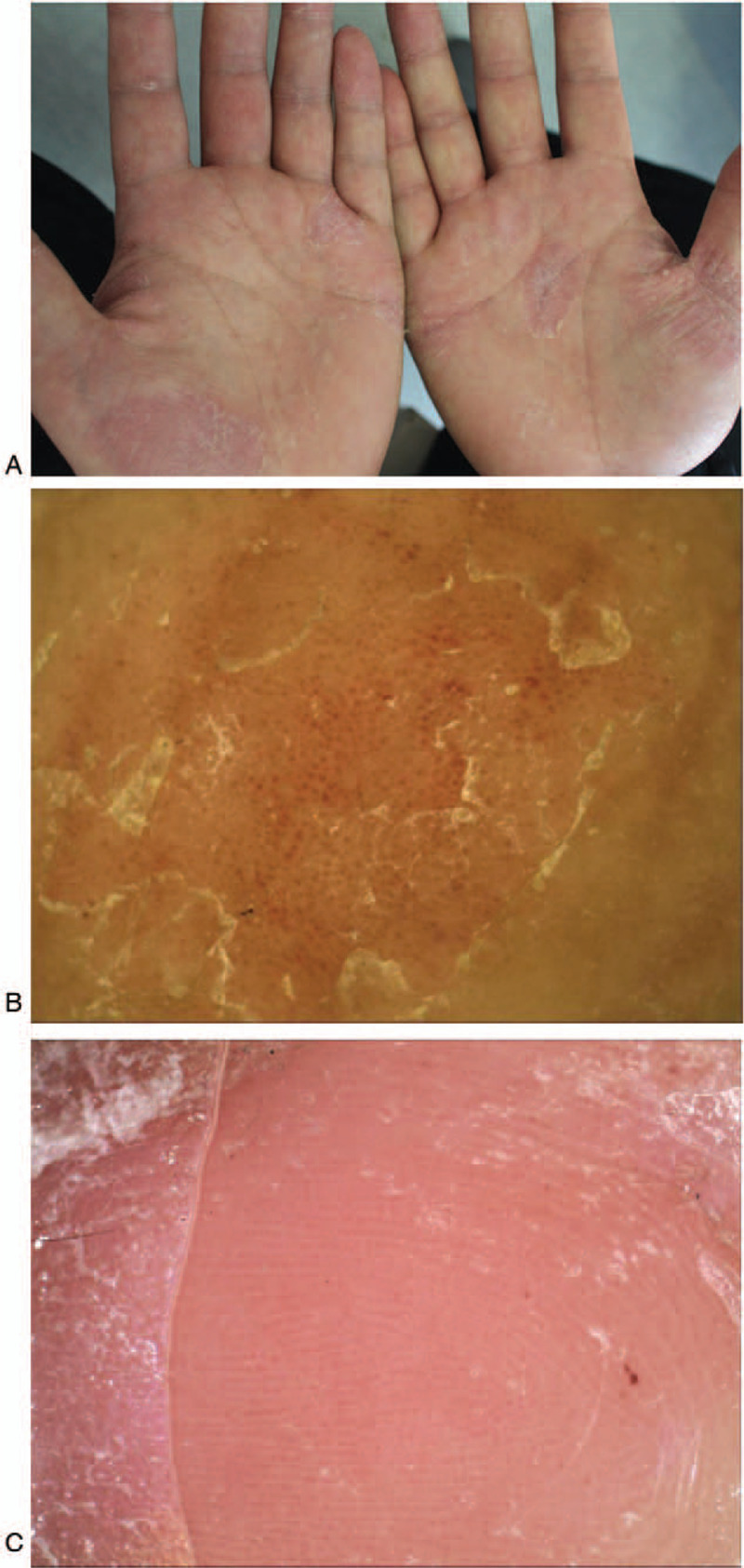
(A) Red patches scattered on the palm, covered with yellow and white scales. (B) Typical dermoscopic appearance of PP, with white scales, regular arrangement of globular vessels (Unpolarized mode ×20). (C) Dotted vessels arranged regularly and distributed along the sulci cutis in partial PP (Unpolarized mode ×20). PP = palmoplantar psoriasis.

**Figure 2:**
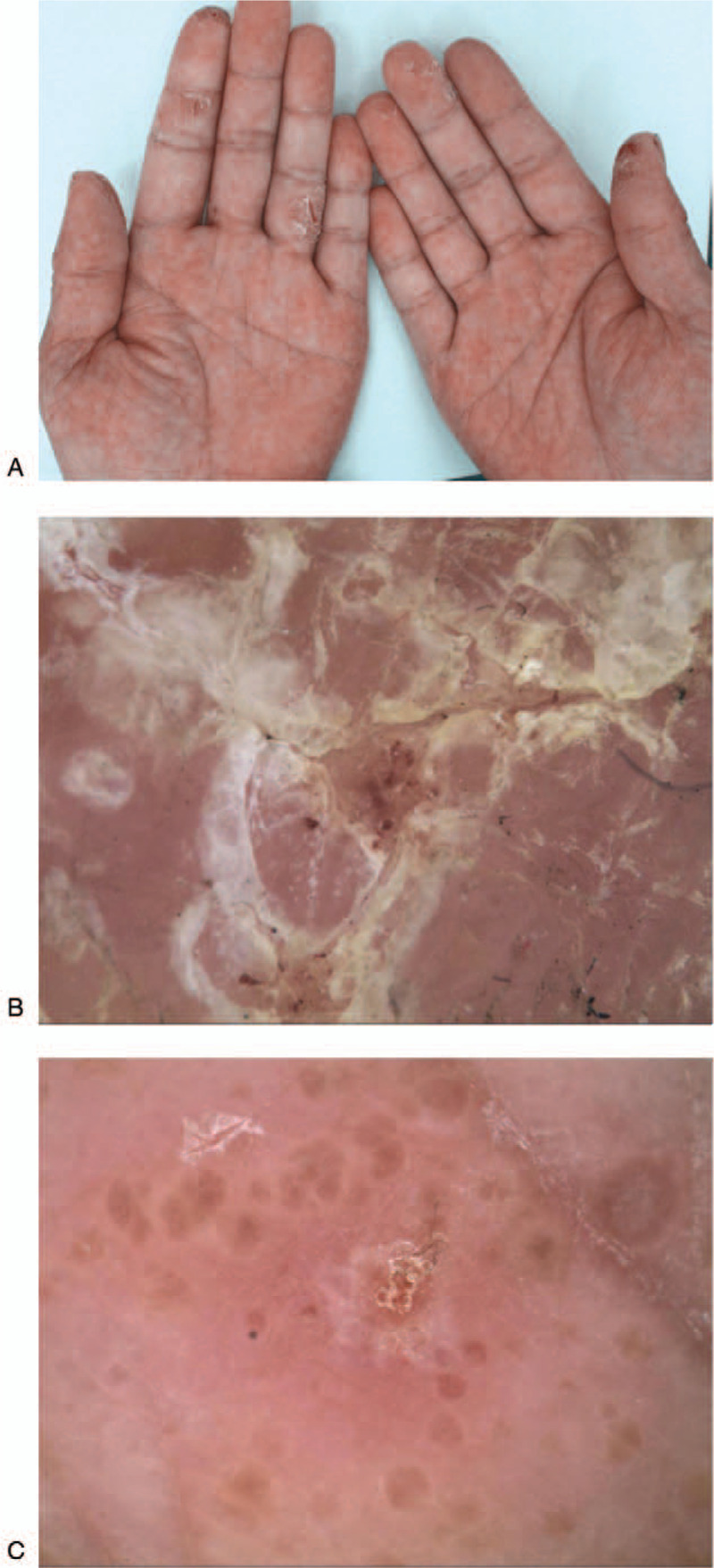
(A) Pink patches scattered on the palm and fingers, covered with yellow and white scales. Cracks visible in certain parts of the skin lesions. (B) The characteristics of PE was yellow scales, irregular arrangement of atypical vessels, and dark red stasis around the cracks (Polarized mode ×20). (C) In some cases of PE, brown-orange-yellow dots/globular structures were observed under the dermatoscope (Unpolarized mode ×20). PE = palmoplantar eczema.

Psoriasis is a chronic inflammatory skin disease that is typically characterized by keratinized plaques with white scales.^[1]^ Eczema is a chronic skin disorder that appears in different forms, including infiltration, edema, vesicles, scaling, and hyperkeratosis accompanied by pruritus.^[2]^ There are obvious differences between psoriasis and eczema in parts of the body, except the palmoplantar. When the skin lesion is limited to the palmoplantar region, palmoplantar psoriasis (PP) (Fig. 1A) can easily be misdiagnosed as palmoplantar eczema (PE) (Fig. 2A) due to the particularity of palmoplantar anatomy and long-term disease history. The pathological characteristics of PP and PE are similar. Both diseases share the pathological features, such as epidermal hyperplasia, parakeratosis, and spongiosis.^[3]^ Additionally, skin biopsy of the palmoplantar region may temporarily affect the quality of life of patients. Therefore, the use of dermoscopy, a non-invasive diagnostic technique, is of great value for distinguishing between PP and PE. There was only 1 previous article that had reported of this type of a study,^[4]^ but their sample size was relatively small. As the number of cases on which we collected data increased, we obtained novel findings.

### 1.2 Objectives

This study aimed to characterize PP and PE by comparing the scales, vascular morphology, vascular distribution of the palmoplantar of PP and PE patients under a dermoscope.

## 2 Methods

Twenty-six patients with psoriasis on the palmoplantar region and 31 patients with eczema on the palmoplantar region were enrolled. The study has been ethically approved by the research institution and the informed consent has been obtained from all patients. The diagnosis of all patients enrolled were pathologically or clinically confirmed. Target palmoplantar areas were observed through general observation and under a Dr. CAMSCOPE dermoscope (Dermat Company, China) with polarized light observations (20 times, 50 times per lens). When the skin lesion was too thick, the scale was removed for other indications to be observed. Dermoscopic features (scale color, vascular patterns under a low power lens, and vascular morphology under a high power lens) were reviewed by a dermoscopist who was not aware of the final diagnosis. All statistical analyses were performed using IBM SPSS Statistics version 19.0 software. The Chi-square test or Fisher test was used for comparison between groups. Differences were considered to be significant at a *P*-value of <.01. Diagnostic tests were used to determine the specificity and sensitivity of the indicators. Youden index, which determines the overall ability of the diagnostic methods in differentiating between patients and those who are not, was calculated using the following formula: sensitivity + specificity – 1. The higher the value of Youden index, the better the diagnostic test and the higher it's level of authenticity. The study was approved by the Review Board and Ethics Committee of the Second Hospital of Shandong University.

## 3 Results

We found that the most common dermoscopic appearance of PP was a red background, white scales, and dot/globular/hairpin-type vessels in a regular arrangement (Fig. 1B), while the presence of pink background, yellow scales, and atypical blood vessels in an irregular arrangement were observed in PE patients (Table 1).

**Table 1:** Dermatoscopic features of palmoplantar psoriasis and palmoplantar eczema.

	Palmoplantar psoriasisn = 26 (%)	Palmoplantar eczeman = 31 (%)	*χ*^*2*^	*P*
Background color				
Red	15 (57.7)	4 (12.9)	12.77	<.001
Pink	11 (42.3)	27 (87.1)	12.77	<.001
Scale color				
White	24 (92.3)	14 (45.2)	14.14	<.001
Yellow	13 (50.0)	19 (61.3)	0.73	.432
Morphology of blood vessels				
Dots or globular vessels	22 (84.6)	7 (22.6)	21.77	<.001
Atypical blood vessels	2 (7.8)	29 (93.5)	42.02	<.001
Hairpin type vessels	9 (34.6)	0 (0)	10.27	<.001
Distribution/arrangement of blood vessels				
Regular arrangement	26 (100)	2 (6.5)	49.51	<.001
Irregular arrangement	1 (3.8)	29 (93.5)	45.64	<.001

The characteristics of PP used for diagnosis under a dermoscope were ranked in the order of the specificity: hairpin type vessels (100%), regular arrangement of blood vessels (93.55%), red background color (87.1%), dotted or globular vessels (77.42%), and white scales (54.84%), of which the Youden Index was highest for an regular arrangement of vessels (0.9355), indicating that this characteristic was the most valuable for the diagnosis (Table 2).

**Table 2:** Sensitivity, specificity, and Youden index of some dermatoscopic features in the diagnosis of psoriasis.

Dermoscopic feature	Sensitivity (%)	Specificity (%)	Youden index
Red background color	57.69	87.10	0.4479
White scale	92.31	54.84	0.4715
Dotted or globular vessel	84.62	77.42	0.6204
Hairpin type vessel	34.62	100.00	0.3462
Regular arrangement of blood vessels	100.00	93.55	0.9355

In addition, we also identified certain special characteristics that cannot always be found but which are very specific. Dotted vessels were found to be distributed in a beaded pattern along the sulci cutis in 8 cases of PP (Fig. 1C). Atypical vessels and dark red stasis can often be observed around cracks (Fig. 2B). Also, brown-orange-yellow dots and globules were found to be significant for the diagnosis of PE (Fig. 2C).

## 4 Discussion

At present, the application of dermoscopy is growing and has resulted in the ability to conduct more in-depth research in the field of inflammatory skin diseases.^[5,6]^ However, there are only a few studies that have used dermoscopy to conduct research on inflammatory skin diseases in the palm. The transparent layer under the cuticle layer causes the skin in the palmoplantar region to be thicker than that of other parts of the body, which leads to difficulties in conducting observations under a dermoscope.

The most characteristic dermoscopic indications of psoriasis is the regular arrangement of hairpin/dots/globular type vessels. This type of vascular morphology is mainly caused by pathological particularity and dot/globular type vessels can be observed when the dermoscope is placed perpendicular to dilated capillaries of the dermal papilla, while ring/hairpin type vessels can be observed when viewed at an angle.

Some studies have found that the probability of observing dot type vessels in PP patients under a dermoscope to be 90% (lower than other parts of the body),^[7]^ which was consistent with our observational results (84.6%). Similarly, hairpin type vessels were found in only 34.6% of PP patients in our study. However, hairpin type vessels still showed a high diagnostic specificity for the diagnosis of psoriasis. Unlike differences between psoriasis and eczema in other parts of the body, the color of scales cannot be used as the key differentiator between PP and PE, which is different from the results obtained by Enzo.^[4]^ The color of PP scales is often similar that of PE scales due to the presence of a thicker corneous layer or local external use of drugs and other factors. PE scales were found to be mostly yellow under the dermoscope due to its main pathological manifestations (irregular hyperplasia of the spinous layer, sponge edema of different degrees, and serous exudation of the cuticle layer).

The new findings in the study may have revealed great value in the differential diagnosis of PP and PE. Dotted vessels distributed in a beaded pattern along the sulci cutis are important indicators for the diagnosis of PP under a dermoscope. Atypical vessels and dark red stasis around cracks observed from PE patients may be related to scratches caused by itching. Brown-orange-yellow dots and globules were found to be significant for the diagnosis of PE, which are histopathological utricle bubbles in the tiny grassroots of skin edema (spongiotic vesicles) due to high levels of resistance in the palmoplantar region.^[8]^ These new findings may lead to a higher clinical implementation of dermoscopy in the differential diagnosis of PP and PE.

In conclusion, dermoscopy is an auxiliary form of examination that was shown to be valuable for the differential diagnosis of psoriasis and chronic eczema in the palmoplantar region, which do not show differences in clinical features. However, correlations between dermoscopic findings and pathological features need to be studied further.

## Author contributions

**Conceptualization:** Xiaoping Yu, Guo Wei, Xiaojie Zhang.

**Data curation:** Xiaoping Yu, Chunchun Shao.

**Formal analysis:** Chunchun Shao, Mingjie Zhu.

**Project administration:** Xiaojie Zhang.

**Resources:** Shuna Sun.

**Supervision:** Xiaojie Zhang.

**Investigation:** Xiaoping Yu.

**Methodology:** Xiaoping Yu, Chunchun Shao, Mingjie Zhu, Xiaojie Zhang.

**Visualization:** Xiaoping Yu.

**Writing – original draft:** Xiaoping Yu, Mingjie Zhu.

**Writing – review & editing:** Guo Wei, Shuna Sun, Xiaojie Zhang.
